# Successful Endoscopic Removal of an Ingested Thumbtack Stuck in the Ileocecal Valve in a Patient With a Psychiatric Disorder

**DOI:** 10.7759/cureus.74798

**Published:** 2024-11-29

**Authors:** Shimpei Asada, Koji Morishita, Shusuke Mori

**Affiliations:** 1 Department of Acute Critical Care and Disaster Medicine, Institute of Science Tokyo, Tokyo, JPN; 2 Department of Emergency Medicine, Tokyo Women's Medical University, Tokyo, JPN

**Keywords:** endoscopic removal, foreign body ingestion, ileocecal valve, mental disorders, thumbtack

## Abstract

Foreign body ingestion is sometimes missed during the initial evaluation of a patient with a psychiatric disorder in the emergency department. This is often due to a lack of awareness regarding the need for thorough physical and diagnostic imaging examinations. Additionally, the management of ingested foreign bodies is often controversial. It is essential to consider the risk of complications, especially with sharp objects in luminal organs, necessitating a cautious and attentive approach to the extraction strategy.

A 20-year-old woman with autism spectrum disorder was brought to the emergency department after being found collapsed. Her consciousness improved upon arrival, and no abnormalities were noted except for mild epigastric tenderness. Based on her regular physical examination, blood tests, and electrocardiogram, the syncopal episode was attributed to psychological factors. However, the patient's mother provided critical information about missing thumbtacks from the patient’s belongings and mentioned prior episodes of thumbtack ingestion. This information underscored the value of obtaining comprehensive patient history in forming an accurate diagnosis. Imaging studies revealed two thumbtacks in the duodenum and small intestine without signs of free air. Conservative management was chosen, and one thumbtack was naturally excreted on the fifth day, while the other remained stuck in the terminal ileum. A subsequent colonoscopy showed the tip of the thumbtack in the ileocecal valve’s lumen, which was successfully removed with forceps without complication.

Endoscopic removal of foreign bodies, being noninvasive, should be the first choice as long as it can reach the target. This technique minimizes patient discomfort and recovery time, instilling confidence in the medical team's management strategy. Furthermore, patients with mental health disorders or dementia, even when specific symptoms are absent, should be regarded as potentially at risk for incidental or unrelated medical conditions. Emergency physicians must maintain a high index of suspicion during initial evaluations. Gathering a comprehensive medical history, including prior behavioral patterns and habitual tendencies, is essential for accurate assessment and management.

## Introduction

Foreign body ingestion, often associated with conditions such as dementia and mental disorders, can lead to serious complications, including perforation, obstruction, and fistula formation [1]. Recent statistics from the United States indicate that the incidence of foreign body ingestion among adults has nearly doubled over the past two decades. Intentional ingestion accounts for approximately 14% of cases, with a rising trend over time. These instances are more commonly observed in younger individuals and are often linked to mental health disorders, cognitive impairments, or psychiatric illnesses, as opposed to accidental ingestions [2].

In cases of sharp object ingestion, gastrointestinal perforation is a potential risk, necessitating the removal of the foreign body. While endoscopic or surgical extraction is frequently considered, conservative management with observation, allowing for natural passage, can sometimes be successful [3]. Approximately 80-90% of ingested foreign bodies are naturally excreted without intervention, while 10-20% require removal via endoscopy or surgery [4]. However, early endoscopic or surgical intervention should be considered based on the shape and nature of the ingested foreign body, especially when natural excretion through stool is unlikely. Sharp objects, in particular, are associated with a 35% perforation rate and impactions, most commonly occurring at the ileocecal valve. Evidence on the role of colonoscopy in managing foreign bodies that have migrated distally into the ileum or colon remains limited [5]. This report details a case of a patient with autism spectrum disorder who ingested thumbtacks and was treated conservatively at our facility. The patient's condition improved without complications, and the foreign body was successfully removed via colonoscopy, demonstrating the effectiveness of this approach.

## Case presentation

A 20-year-old woman with a history of autism spectrum disorder and multiple episodes of consciousness loss due to dissociative consciousness disturbances was brought to our department after being found collapsed in a store restroom. By the time of arrival, her consciousness had improved to full alertness. Although no abnormalities were noted in her vital signs at arrival, there was mild tenderness in the epigastric region. The initial differential diagnosis for the patient's loss of consciousness included an underlying psychiatric disorder, given her history of previous syncopal episodes, and a vasovagal reflex, as she exhibited no laboratory or neurological abnormalities and experienced a rapid recovery from the syncopal event. Secondary considerations included gastroenteritis, gastroesophageal reflux disease, or peptic ulcers, given the presence of mild, albeit vague and non-reproducible, epigastric tenderness. Despite an exhaustive evaluation, including physical examination, blood tests, and electrocardiogram, no definitive cause for the loss of consciousness was identified.

**Figure 1:**
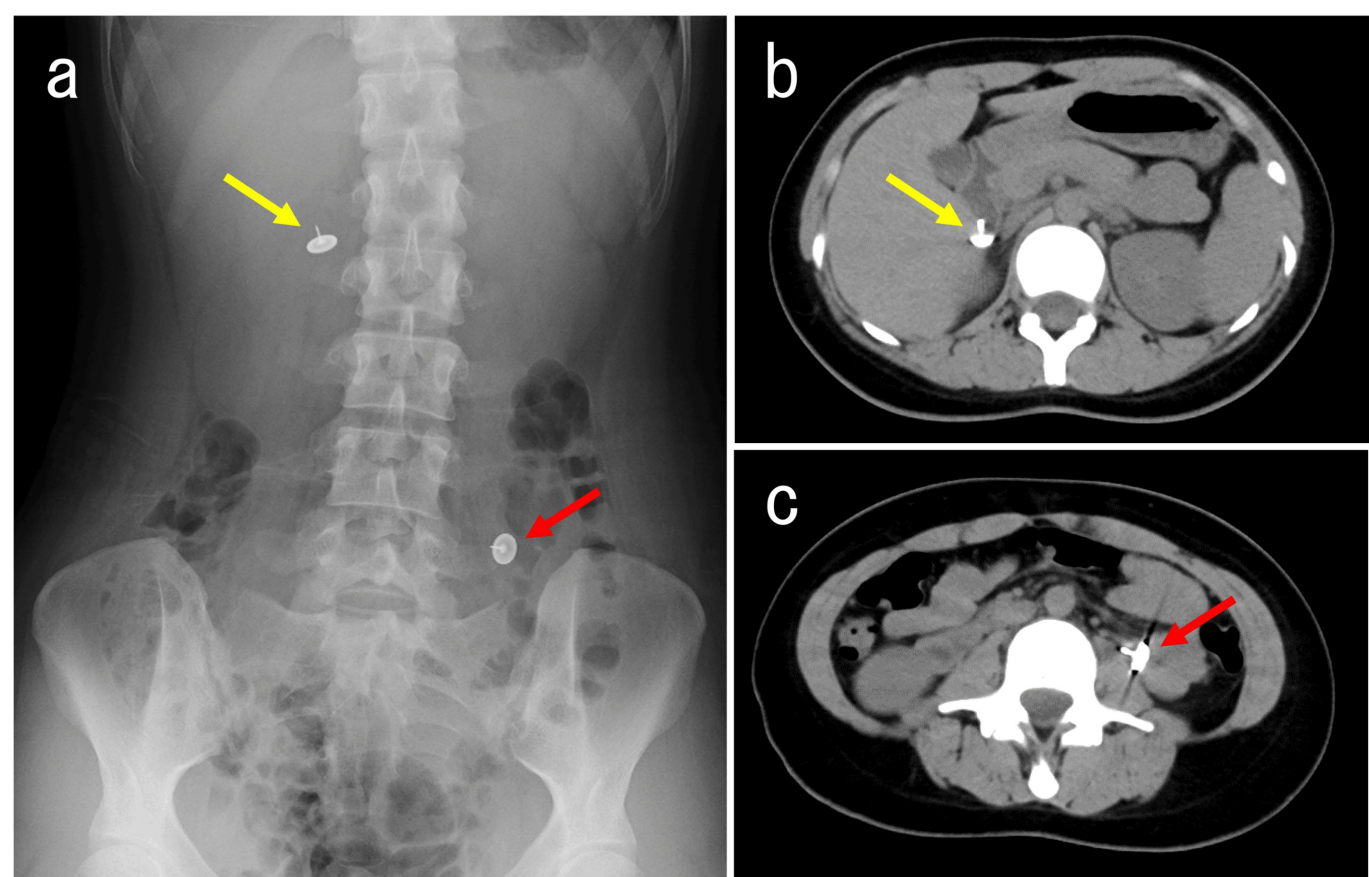
An abdominal X-ray and computed tomography imaging (a) An initial abdominal X-ray revealed two thumbtacks: one in the right upper abdomen (yellow arrow) and the other in the left lower abdomen (red arrow). (b, c) CT scans were performed to accurately locate the foreign bodies and rule out complications such as perforation. The scans identified one thumbtack in the descending duodenum (yellow arrow) and the other in the proximal jejunum (red arrow). No evidence of free air suggestive of gastrointestinal perforation was detected. Endoscopic retrieval was deemed infeasible, and conservative management was chosen.

Despite the lack of explanatory causes found through physical examination, blood tests, and electrocardiogram, our team conducted a thorough evaluation. Given her history, it was strongly suspected that the loss of consciousness was due to psychological factors. Although discharge from the emergency department was being considered, the accompanying mother pointed out the presence of an unusually placed thumbtack among the patient's belongings and mentioned that the patient had previously ingested thumbtacks for self-harm purposes. It was noted that during a previous incident of thumbtack ingestion, the patient had been hospitalized at another facility, and the thumbtacks were naturally excreted. To investigate the possibility of foreign body ingestion, an abdominal X-ray was performed based on the provided information, revealing the presence of two thumbtacks. Following confirmation of the ingestion, a computed tomography (CT) scan was conducted to precisely determine the thumbtacks' locations and rule out free air indicative of gastrointestinal perforation. The CT scan identified one thumbtack in the descending duodenum and another in the proximal small intestine. No free air suggestive of perforation was observed on imaging (Figure 1).

**Figure 2:**
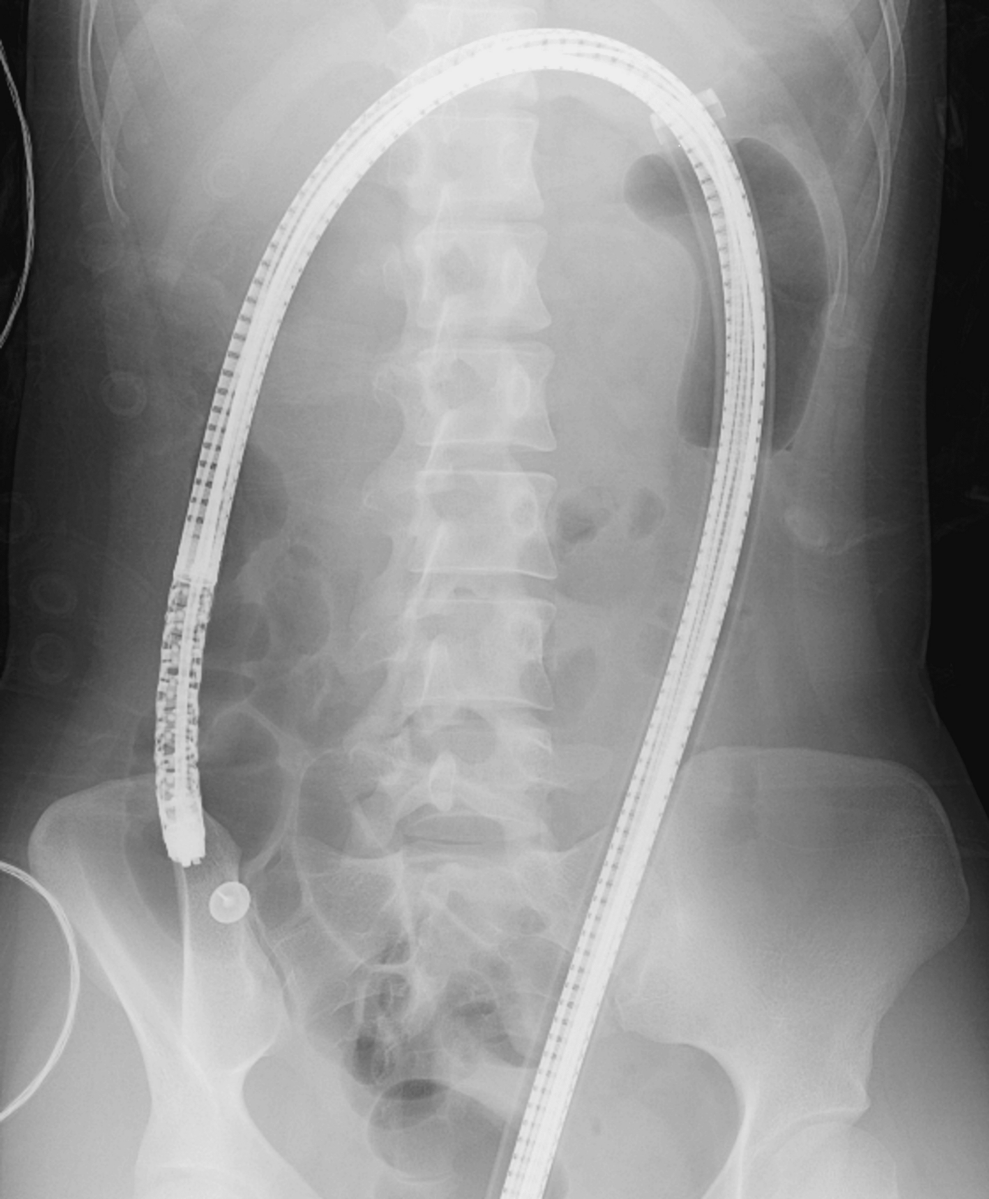
Fluoroscopy performed during colonoscopy Colonoscopy, performed under fluoroscopic guidance, successfully reached the cecum. The tip of the endoscope was visualized near the retained thumbtacks at the ileocecal valve. These findings were crucial in the decision to proceed with endoscopic removal.

Endoscopic removal was deemed difficult, so, given the absence of perforation, conservative management with the expectation of natural passage was chosen, and the patient was admitted for observation under fasting. During hospitalization, the patient did not experience worsening abdominal pain. Daily abdominal X-rays were taken, and by the third hospital day, both thumbtacks had migrated to the area between the cecum and ascending colon. One thumbtack was excreted on the fifth hospital day, but the other remained in the ileocecal region. A lower gastrointestinal endoscopy was performed on the same day (Figure 2).

**Figure 3:**
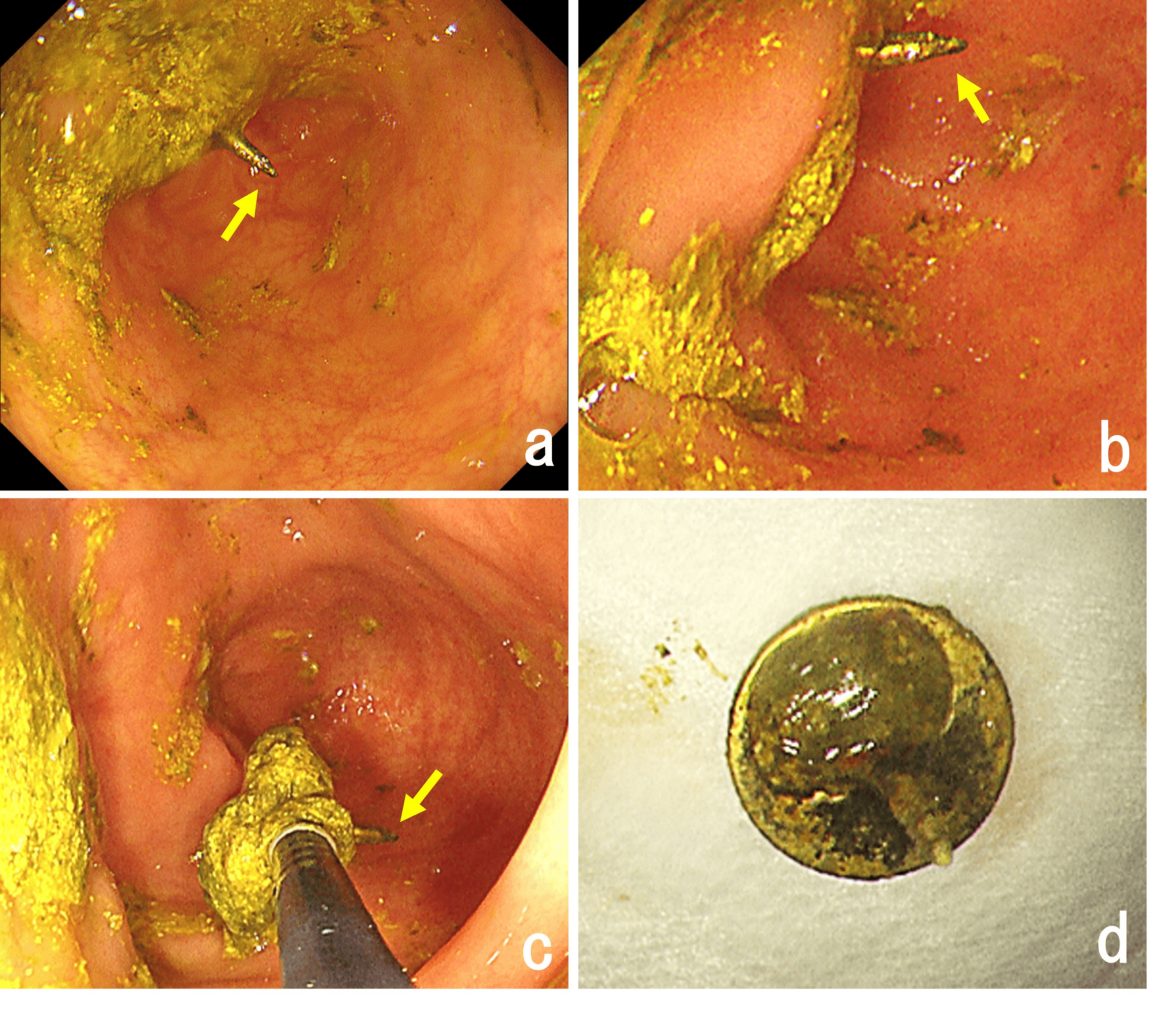
Colonoscopic images showing the process of removing the thumbtack (a, b) Colonoscopic images revealed a thumbtack lodged in the lumen of the ileocecal valve, with the sharp end projecting outward (arrows). (c) The tip of the thumbtack (arrow) was securely grasped with forceps and successfully extracted from the ileocecal valve’s lumen without any adverse events. (d) The extracted thumbtack is shown.

The procedure was conducted using an external sheath. Upon examining the cecum, the tip of the thumbtack was found embedded in the ileocecal valve. It was grasped with forceps, extracted into the sheath, and removed. No mucosal damage was noted, and treatment was concluded (Figure 3). The patient resumed eating the following day and was discharged on the same day after confirming no changes in symptoms. During the patient’s admission, the psychiatry liaison team was consulted to provide psychiatric care. A follow-up plan was also established for her discharge, including a referral letter to her family psychiatrist, who managed ongoing psychotherapy and medication prescriptions. This successful outcome underscores the effectiveness of the chosen management strategy.

## Discussion

Foreign body ingestion results in the death of approximately 1,500 people annually in the United States [6]. Most cases of foreign body ingestion in pediatrics are unintentional, while in adults, intentional or accidental ingestion is often associated with mental illness or dementia [2]. It can occur for various reasons, including pica, acuphagia, suicidal intent, and accidental ingestion. Pica and acuphagia are classified as eating disorders and are often associated with mental illnesses such as dementia and other psychiatric disorders [1,7,8]. Patients frequently present with a wide range of symptoms or complaints, making it challenging for physicians to suspect foreign body ingestion in the emergency department. A variety of signs and symptoms have been reported following the ingestion or aspiration of foreign bodies, with approximately half of those who ingest foreign bodies remaining asymptomatic [9]. Immediate clinical manifestations of foreign body ingestion range from epigastric pain (55%), vomiting (16%), dysphagia (7%), pharyngeal discomfort (4%), and chest pain (3%) to the absence of symptoms (30%) [9]. Therefore, obtaining a thorough medical history and understanding the patient’s habits, either from the patient themselves or from their accompanying guardians, is crucial to avoid missing incidental cases of foreign body ingestion [8].

In this case, the initial reason for the patient's visit was a loss of consciousness, complicating the suspicion of foreign body ingestion. However, through comprehensive history-taking, gathering information from accompanying individuals, and conducting a careful abdominal examination, we were led to suspect foreign body ingestion and were able to make an accurate diagnosis. This highlights the importance of special attention for patients with mental disorders or dementia. Evaluating the causal relationship between loss of consciousness and foreign body ingestion can be challenging, but a thorough examination of all aspects of the medical history, background, and physical findings is essential [8,10].

Gastrointestinal foreign bodies are generally expected to be expelled through conservative management; however, sharp objects, button batteries, and large items that are likely to be impassible may require endoscopic or surgical removal [11]. Additionally, objects that remain in the gastrointestinal tract for an extended period can result in obstruction or ulcer formation, potentially leading to perforation [12]. The management of ingested foreign bodies remains a subject of ongoing debate. A retrospective analysis of 542 cases of foreign body ingestion found that 75.6% of foreign bodies passed successfully, 19.5% required endoscopic removal, and 4.8% necessitated surgery [13]. The conventional methods for monitoring location and complications, such as perforation and obstruction, primarily include radiographs and computed tomography (CT). X-rays are easy to perform and can be repeated, but they may not always accurately detect the exact location of the foreign body or complications. Additionally, certain materials are radiolucent and difficult to identify. In contrast, CT offers the advantage of precisely locating the foreign body, even when it is less radiopaque, while also allowing for controlled radiation dosage. These two imaging modalities are essential for monitoring ingested foreign bodies [5].

Risk factors for complications include sharp objects, items larger than 6 mm, recurrent ingestion, and a history of gastrointestinal tract surgeries. Sharp objects are responsible for 35% of perforations and impactions, most commonly at the ileocecal valve [14]. According to the current ASGE guidelines, sharp objects located distal to the ligament of Treitz are typically managed conservatively with serial abdominal examinations, a bowel regimen, and stool monitoring for the passage of the foreign body. The guidelines also recommend close clinical monitoring for signs of bowel obstruction or perforation. Surgical or endoscopic removal is indicated if the foreign body fails to pass within three to five days. However, no specific ASGE guidelines exist for colonoscopic removal of foreign bodies that fail conservative management. Based on a review of the literature and our case, early colonoscopy is recommended as a safe and effective approach for removing accessible sharp objects, reducing the risk of perforation and the need for exploratory surgery [14-16]. In this instance, the sharp end of the thumbtack was protruding into the cecum, making observation, identification, and removal straightforward.

The timing of the colonoscopy, performed after five days of careful observation, was appropriate and in line with current guidelines. When deciding between endoscopic and surgical interventions, the former is preferred due to its lower invasiveness. However, endoscopic observation or retrieval becomes challenging when the foreign body is located beyond the horizontal portion of the duodenum, extending just proximal to the ileocecal valve, in which case surgery is preferred [13]. In cases of prolonged foreign body retention in the ileocecal valve, such as in this case, there is a risk of intestinal obstruction and perforation, necessitating prompt removal. In these situations, colonoscopy is preferred over surgery due to its less invasive nature [17].

## Conclusions

Ingested foreign bodies can lead to various complications, making precise diagnosis and vigilant management essential upon admission. Many cases of foreign body ingestion are symptomatic, but in patients with mental disorders or dementia, symptoms may be minimal or absent. In such cases, obtaining a thorough medical history or gathering information about previous behavioral or habitual episodes is often crucial. Close monitoring with X-ray or CT imaging is recommended if the foreign body has passed beyond the small intestine. If the foreign body remains in the gastrointestinal tract for five days or more, endoscopic or surgical removal should be considered due to the risk of complications such as perforation and obstruction. In cases where the foreign body is lodged in the ileocecal valve, endoscopic removal is a non-invasive and rapid procedure, making it the preferred approach as long as it is accessible. Additionally, psychiatric care, including collaboration with a liaison team and the family psychiatrist, is essential to prevent the recurrence of such episodes.
